# Immune-related adverse events and clinical outcomes in advanced non-small cell lung cancer: does more toxicity predict better outcomes?

**DOI:** 10.3389/fimmu.2026.1947761

**Published:** 2026-09-17

**Authors:** Abed Agbarya, Noa Shani Shrem, Asmah Miari, Ronen Brenner, Yulia Dudnik, Natalie Maimon Rabinovich, Amichay Meirovitz, Alexander Yakobson, Ahron Yehonatan Cohen, Nashat Abu Yasin, Adan Khalaily, Raya Bdair, Mahmoud Abu Amna, Walid Shalata

**Affiliations:** 1 Department of Oncology, Bnai Zion Medical Center, Haifa, Israel; 2 The Legacy Heritage Cancer Center, Larry Norton Institute, Soroka Medical Center, Beer Sheva, Israel; 3 Faculty of Health Sciences, Ben Gurion University of the Negev, Beer Sheva, Israel; 4 Department of Oncology, Meir Medical Center, Kfar Saba, Israel; 5 Edith Wolfson Medical Center, Oncology Institute, Holon, Israel; 6 Department of Oncology, Emek Medical Centre, Afula, Israel

**Keywords:** age-related differences, immune checkpoint inhibitors, immune-related adverse events, non–small cell lung cancer, overall survival, progression-free survival

## Abstract

**Background:**

Immune checkpoint inhibitors (ICIs) are a cornerstone of therapy for advanced non–small cell lung cancer (NSCLC). Elderly patients remain underrepresented in clinical trials, and the prognostic significance of immune-related adverse events (irAEs)—including their cumulative burden—across age groups is not fully defined. This study evaluated age-related differences in clinical characteristics, treatment patterns, irAEs, and outcomes in patients with stage IV NSCLC.

**Methods:**

This retrospective multicenter study included 452 patients with metastatic NSCLC treated with ICIs, with or without chemotherapy. Patients were stratified by age (<75 vs. ≥75 years). irAEs were analyzed both by occurrence (yes vs. no) and by burden (≤2 vs. >2 different irAE types). Progression-free survival (PFS) and overall survival (OS) were estimated using the Kaplan–Meier method and compared across age and irAE subgroups.

**Results:**

Among the 452 patients, 344 (76.1%) were <75 years old and 108 (23.9%) were of ages ≥75 years. Baseline characteristics, including histology and PD-L1 expression, were comparable between age groups. Older patients were more frequently treated with immunotherapy alone or dual immunotherapy and less often received chemo-immunotherapy (p = 0.024). The distribution of irAEs differed significantly by age (p < 0.001), with patients ≥75 years of age experiencing a higher frequency of >2 irAE types. The median PFS and OS for the entire cohort were 12.0 and 15.0 months, respectively. While PFS was similar between age groups, OS was significantly shorter in patients of age ≥75 years (p = 0.008). Across both age groups, the occurrence of irAEs was associated with significantly improved PFS and OS. A higher irAE burden (>2 different types) was associated with numerically longer survival in both younger and older patients.

**Conclusions:**

ICIs are effective in elderly patients with advanced NSCLC. The presence of irAEs—and potentially a higher irAE burden—is associated with improved survival outcomes regardless of age, supporting irAEs as a clinically meaningful biomarker of immunotherapy benefit, including in patients ≥75 years of age.

## 1 Introduction

Lung cancer remains the primary cause of cancer-related mortality worldwide, accounting for approximately 1.8 million deaths annually (1). Histologically, it is broadly categorized into two major subtypes: non-small cell lung cancer (NSCLC) and small cell lung cancer (SCLC). NSCLC represents approximately 85% of all lung cancer diagnoses, whereas SCLC accounts for the remaining 15% (2). NSCLC is further stratified into three predominant histological subtypes: adenocarcinoma, the most common variant, which typically arises in the distal airways; squamous cell carcinoma, which predominantly develops in the central airways; and large cell carcinoma, an undifferentiated subtype diagnosed by exclusion when tumors lack distinct glandular or squamous features (3).

The therapeutic paradigm for NSCLC has been radically transformed by the clinical introduction of immune checkpoint inhibitors (ICIs)—specifically agents targeting the programed cell death-1 (PD-1), programed cell death-ligand 1 (PD-L1), and cytotoxic T-lymphocyte-associated protein 4 (CTLA-4) pathways (4). By interrupting these inhibitory signals, ICIs restore the host immune system’s ability to recognize and eradicate malignant cells, yielding durable anti-tumor responses that significantly surpass traditional platinum-based chemotherapy (5, 6).

Despite these clinical advances, widespread immune activation can induce a unique spectrum of toxicities termed immune-related adverse events (irAEs), which stem from inflammatory damage to healthy tissues such as the skin, gastrointestinal tract, and lungs (7, 8). These irAEs present with diverse clinical manifestations. While common mild-to-moderate events include fatigue, pruritus, nausea, rash, and endocrinopathies (primarily thyroiditis and hypophysitis), clinicians must remain vigilant for severe, high-grade complications. Among these, pneumonitis and colitis represent the most frequent high-grade irAEs, whereas myocarditis—though less common—carries the highest mortality rate (9).

Checkpoint inhibitor–associated pneumonitis (CIP) is of paramount clinical concern due to its substantial morbidity, standing as the primary cause of irAE-related fatalities in patients receiving anti–PD-1/PD-L1 monotherapy (10). Additional manifestations—such as hepatitis, musculoskeletal symptoms (including myalgias and arthralgias), and less frequently, renal and neurological involvement—have also been well-documented (11).

Understanding the relationship between patient age and irAE severity is critical for clinical decision-making, particularly as older individuals frequently present with immunosenescence and multiple comorbidities. However, current evidence indicates that chronological age alone does not correlate with a higher incidence of severe toxicities; specifically, the frequency of Grade 3–5 irAEs—those requiring systemic corticosteroids or permanent treatment discontinuation—remains remarkably similar between older (≥65 years) and younger patients (12, 13).

Central to this comparison is the emerging “toxicity-efficacy” paradigm, which posits that irAE development is not merely an untoward complication, but rather a surrogate biomarker of robust anti-tumor immunity. This hypothesis suggests that autoimmune side effects reflect a heightened, active immune response capable of targeting malignant cells with comparable potency. In support of this concept, multiple meta-analysis and systematic reviews demonstrate that the onset of irAEs during ICI therapy correlates with superior treatment response and prolonged overall survival, positioning irAEs as clinical indicators of effective immune engagement (14–16).

Building upon this rationale, this study evaluates whether the frequency and severity of irAEs correlate with superior tumor suppression across distinct age demographics. Specifically, we aim to determine if irAEs serve as a consistent prognostic predictor in both younger and elderly patients, thereby clarifying their utility as a clinical tool for monitoring treatment efficacy.

## 2 Materials and methods

### 2.1 Study population

Data were retrospectively collected from multiple Israeli centers (2020–2025) for metastatic NSCLC patients treated with ICI-based therapies (pembrolizumab or nivolumab/ipilimumab), with or without platinum chemotherapy. To reliably evaluate long-term outcomes and toxicities, the study exclusively enrolled “responders” defined by a PFS of over 4 months.

### 2.2 Study design

The primary objective of this study was to re-evaluate the real-world incidence, spectrum, and severity of irAEs in patients with metastatic NSCLC treated with ICIs. Particular emphasis was placed on age-related differences in toxicity profiles and clinical outcomes. In addition, the study explored whether the burden of irAEs—defined as the occurrence of up to two versus more than two distinct irAE types—could serve as a potential predictive marker for improved treatment response, prolonged PFS, and OS. Patients were stratified according to age at diagnosis (<75 years vs. ≥75 years) to assess whether older adults exhibited differential toxicity patterns, increased clinical consequences, or novel safety signals compared with younger patients. The overarching aim was to optimize risk–benefit assessment of ICI-based therapies in the elderly population. Covariates incorporated into multivariable analyses included the presence and number of irAEs, histological subtype (adenocarcinoma vs. squamous cell carcinoma), sex, age group, smoking status (never, former, or current), Eastern Cooperative Oncology Group performance status (ECOG-PS; 0–1 vs. ≥2), treatment regimen (nivolumab/ipilimumab combination vs. pembrolizumab monotherapy), and tumor PD-L1 expression (<1% vs. ≥1%).

### 2.3 Exclusion criteria

To maintain cohort homogeneity and ensure the integrity of the data, several exclusion criteria were applied. Patients were ineligible if they:

Received chemotherapy without concurrent immunotherapy.

Lacked comprehensive documentation regarding adverse events.

Harbored actionable oncogenic drivers (e.g., EGFR, ALK, ROS1, RET, or BRAF mutations).

Presented with synchronous primary malignancies.

Had a history of prior systemic anticancer therapy, including previous lines of chemotherapy or immunotherapy.

### 2.4 Statistical analysis

Baseline characteristics, including demographic, clinical, and molecular data, were analyzed using descriptive statistics. To account for distribution, continuous variables are reported as medians (ranges), whereas categorical data are expressed as frequencies and percentages. Comparative analyses were stratified by age (<75 vs. ≥ 75 years), irAE burden (≤ 2 vs. > 2 types), and ECOG performance status. Survival curves for PFS and OS were generated using the Kaplan–Meier method and compared via log-rank tests. To identify independent predictors of survival, multivariable Cox proportional hazards regression models were utilized, with results expressed as hazard ratios (HRs) and 95% confidence intervals (CIs). We visualized irAE distributions across age groups using graphical stratification for variables such as PD-L1 expression, treatment regimen, and histology. Statistical significance was defined as a two-sided p < 0.05. All analyses were performed using IBM SPSS Statistics (version 29.0).

## 3 Results

The study included a total of 452 patients with stage 4 NSCLC, of whom 344 (76.1%) were <75 years and 108 (23.9%) were aged ≥ 75 years. The overall median age was 68 years (range 37–87). Gender distribution was similar between age groups, with males representing the majority of the cohort (70.4% overall). Female patients accounted for 29.6% of the entire population, with no statistically significant difference between younger and older patients (29.0% vs. 31.4%, p = 0.72). Histological subtypes were well balanced across age groups. Adenocarcinoma was the predominant histology, observed in 67.9% of the overall cohort, while squamous cell carcinoma accounted for 32.1%. No significant differences in histological distribution were noted (p=0.972) nor was there significant difference in PD-L1 expression between patients in the two age groups. Overall, 40.2% of patients had PD-L1 expression <1%, and 59.8% had PD-L1 expression ≥1%. The proportion of PD-L1–negative tumors was slightly higher in the older group, but this difference did not reach statistical significance (44.4% vs. 38.9%, p = 0.367). Smoking status differed significantly by age. Older patients were more likely to be never-smokers compared with younger patients (21.2% vs. 11.9%), whereas current smoking was less frequent in the ≥75-year group (40.7% vs. 51.4%). This difference in smoking distribution was statistically significant (p = 0.032). Baseline performance status showed a trend toward worse ECOG scores among older patients. ECOG 2 was more frequent in patients ≥75 years compared with younger patients (25.1% vs. 17.0%), although this difference did not reach statistical significance (p = 0.059). Regarding treatment, chemo-immunotherapy was the most commonly administered regimen overall (81.8%). However, older patients were significantly more likely to receive immunotherapy alone compared with younger patients (26.0% vs. 15.7%), while chemo-immunotherapy was less frequently used in the ≥75-year group (p = 0.024). The type of immunotherapy administered also differed by age. Pembrolizumab was the most commonly used agent overall (60.2%), but older patients were more frequently treated with ipilimumab plus nivolumab compared with younger patients (49.0% vs. 36.9%). This difference was statistically significant (p = 0.032). IrAEs showed a significant age-related difference. The absence of irAEs was more common among patients ≥75 years of age compared with those <75 years old (12.9% vs. 22.3%), while a higher proportion of older patients experienced more than two irAEs (29.7% vs. 21.4%). Overall, the distribution of irAEs differed significantly between age groups (p < 0.001). (**Table 1**).

**Table 1:** Patients’ characteristics.

Characteristics	Overall (%), N = 452	Age <75 years N=344	Age >75 years N=108	p value
Age (years)				
Median (range)	68 (37-87)	65 (37-74)	79 (75-87)	<0.001
Gender, n (%)				
Female	134 (29.6)	100 (29)	34 (31.4)	0.72
Male	318 (70.4)	244 (71)	74 (68.6)	
Histology, n (%)				
Adenocarcinoma	307 (67.9)	233 (67.7)	74 (68.5)	0.972
Squamous cell carcinoma	145 (32.1)	111 (32.3)	34 (31.5)	
PDL, n (%)				
PDL <1%	182 (40.2)	134 (38.9)	48 (44.4)	0.367
PDL >1%	270 (59.8)	210 (61.1)	60 (55.6)	
Smoking status, n (%)				
Never	\64 (14.1)	41 (11.9)	23 (21.2)	0.032
Current	221 (48.8)	177 (51.4)	44 (40.7)	
Past	162 (35.8)	121(35.1)	41 (38.1)	
Unknown	5 (1.3)	5 (1.6)	0 (0)	
ECOG, n (%)				
0	125 (27.6)	103 (29.9)	22 (20.3)	0.059
1	242 (53.5)	183 (53.1)	59 (54.6)	
2	85 (18.9)	58 (17)	27 (25.1)	
Type of treatment administrated, n (%)				
Chemo-IO	370 (81.8)	290 (84.3)	80 (74.0)	0.024
IO	82 (18.2)	54 (15.7)	28 (26.0)	
Type of immunotherapy, n (%)				
Ipilimumab plus Nivolumab	180 (39.8)	127 (36.9)	53 (49.0)	0.032
Pembrolizumab	272 (60.2)	217 (63.1)	55 (51.0)	
Immune related adverse events, n (%)				
0	91 (20.1)	77 (22.3)	14 (12.9)	<0.001
1-2	256 (56.7)	194 (56.3)	62 (57.4)	
>2	105 (23.2)	73 (21.4)	32 (29.7)	
Outcome				
Median PFS, months	12	12	11	0.13
Median OS, months	15	15	14	0.008

n, number of patients; PD-L1, Programmed Death-Ligand 1; ECOG, Eastern Cooperative Oncology Group (performance status scale); IO, Immunotherapy; Chemo-IO, Combination chemotherapy and immunotherapy; PFS, Progression-Free Survival; OS, Overall Survival.

Overall, irAEs were frequently observed in the study population. Fatigue was the most common irAE, affecting 65.4% of patients overall, with a comparable incidence between patients <75 years and those ≥75 years of age (63.9% vs. 70.3%, p = 0.88). Cutaneous toxicities were among the most commonly reported irAEs. Rash occurred in 13.9% of patients overall and was slightly more frequent in the older age group (16.6% vs. 13.0%), although this difference was not statistically significant (p = 0.12). Similarly, pruritus was reported in 12.6% of patients, with comparable rates between younger and older patients (11.3% vs. 16.6%, p = 0.89). Musculoskeletal toxicity in the form of arthralgia was observed in 8.8% of the cohort and was significantly more common among patients ≥75 years of age compared with those <75 years old (12.0% vs. 7.8%, p = 0.48). Gastrointestinal and pulmonary irAEs were also commonly observed but did not differ significantly by age. Diarrhea occurred in 13.4% of patients overall (12.7% in patients <75 years vs. 15.7% in those ≥75 years old; p = 0.47), while pneumonitis was reported in 10.6% of the cohort (9.5% vs. 13.8%, p = 0.38). Laboratory abnormalities suggestive of immune-mediated organ involvement were infrequent and showed no statistically significant differences between age groups. Transaminase elevations were observed in 10.1% of patients overall (9.3% vs. 12.9%, p = 0.15), and creatinine elevations in 8.1% (6.3% vs. 13.8%). Endocrine irAEs were relatively uncommon. Hyperthyroidism occurred in 2.8% of patients overall, with similar rates between age groups (2.6% vs. 3.7%), whereas hypothyroidism was reported in 17.0% of patients, occurring more frequently among patients ≥75 years old, although this difference was not statistically significant (21.2% vs. 15.6%, p = 0.24). Infusion-related reactions were rare, occurring in only one patient (0.2%) in the entire cohort. (**Table 2**).

**Table 2:** Patients reported adverse events.

Adverse event, n (%)	Overall N = 452	Age <75 years N=344	Age ≥75 years N = 108	*p* value
Fatigue	296 (65.4)	220 (63.9)	76 (70.3)	0.88
Rash	63 (13.9)	45 (13.0)	18 (16.6)	0.12
Pruritus	57 (12.6)	39 (11.3)	18 (16.6)	0.89
Arthralgia	40 (8.8)	27 (7.8)	13 (12.0)	0.48
Infusion reaction	1 (0.2)	1 (0.2)	0 (0.0)	1
Diarrhea	61 (13.4)	44 (12.7)	17 (15.7)	0.47
Pneumonitis	48 (10.6)	33 (9.5)	15 (13.8)	0.38
Transaminase elevation	46 (10.1)	32 (9.3)	14 (12.9)	0.15
Creatinine elevation	37 (8.1)	22 (6.3)	15 (13.8)	0.053
Hyperthyroidism	13 (2.8)	9 (2.6)	4 (3.7)	—
Hypothyroidism	77 (17.0)	54 (15.6)	23 (21.2)	0.24

n, number of patients.

For the entire cohort, the median PFS was 12.0 months, and the median OS was 15.0 months (**Figures 1A, B**). Age-stratified analysis revealed that while younger patients (<75 years) had a slightly longer median PFS than their older counterparts (≥75 years)—12 vs. 11 months, respectively—this trend did not achieve statistical significance (p = 0.13). However, a significant age-related disparity was noted in OS (p = 0.008); patients >75 years had a median OS of 14 months, compared to 15 months for those <75 years.

**Figure 1:**
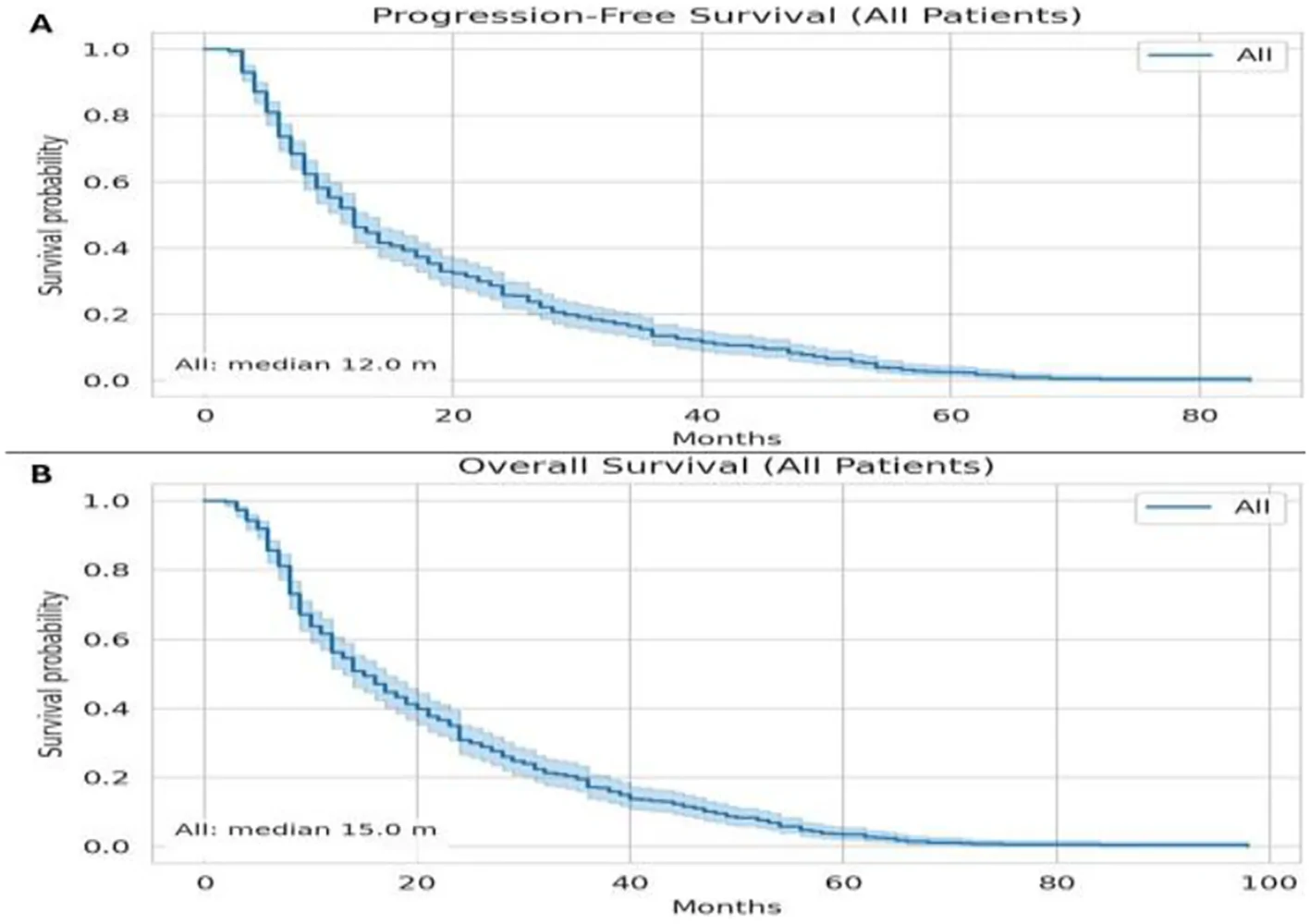
**(A, B)** The progression - free survival was 12 months, and the overall - survival was 15 months for entire cohort.

Among patients <75 years (**Figure 2A**), the occurrence of at least one adverse event was associated with a significantly longer median PFS compared to patients without adverse events (12.0 vs. 10.0 months; p = 0.03). Similarly, in patients ≥75 years old (**Figure 2B**), the effect of irAEs was even more pronounced, with median PFS doubling from 6.0 to 12.0 months in the presence of an irAE (p = 0.05).

**Figure 2:**
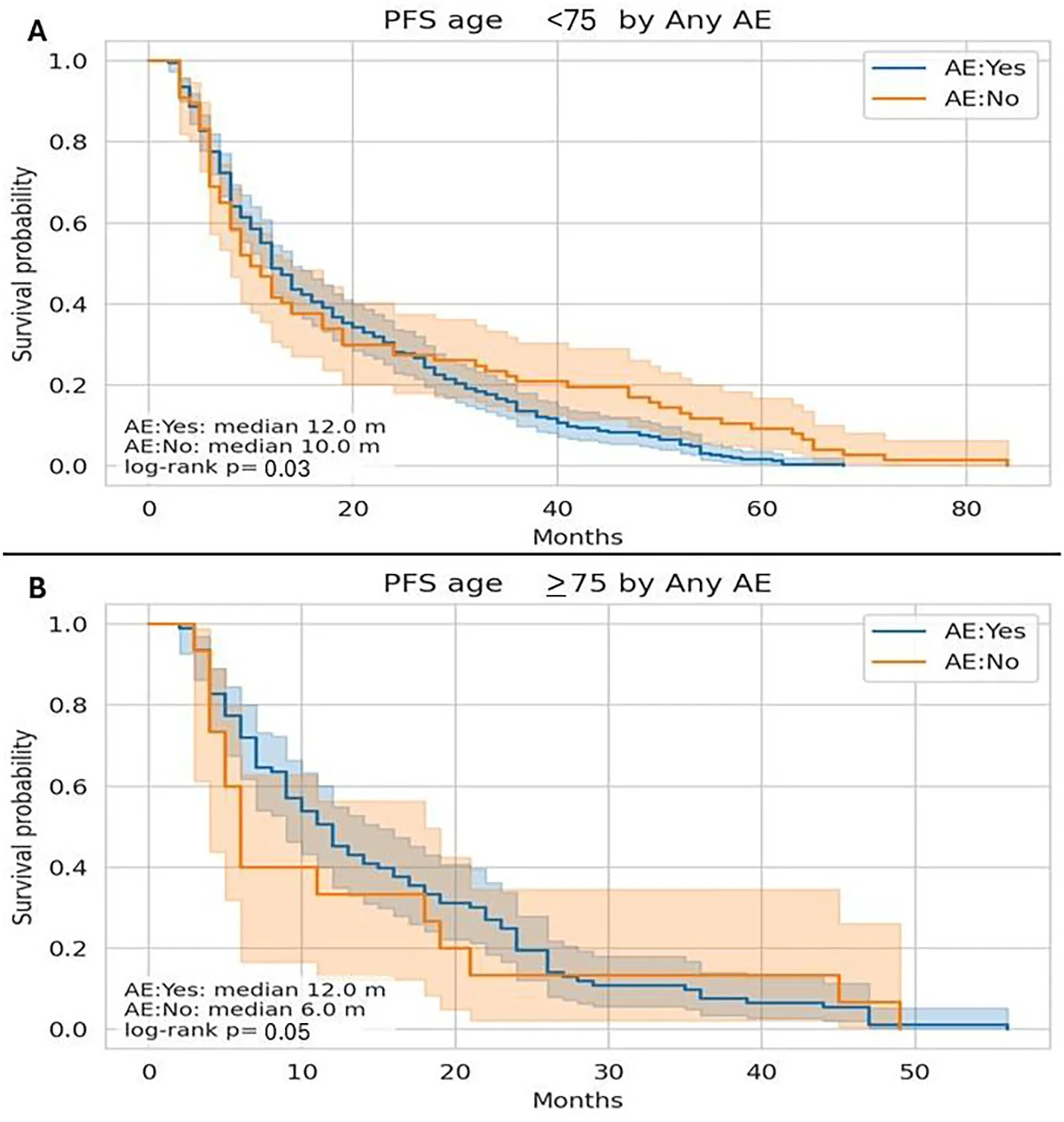
Progression-free survival (PFS) according to the occurrence of immune-related adverse events (irAEs), stratified by age (<75 vs. ≥75 years). **(A)** Among patients aged <75 years, median PFS was 12 months in those who developed irAEs compared with 10 months in those without irAEs (p = 0.03). **(B)** Among patients aged ≥75 years, median PFS was 12 months in patients with irAEs versus 6 months in those without irAEs (p = 0.05).

Among patients ≥75 years of age (**Figure 3A**), a higher number of adverse events was associated with a numerical increase in median PFS from 10.0 to 12.0 months, although this did not reach statistical significance (p = 0.09). Similarly, in patients <75 years (**Figure 3B**), those with more than two adverse events had a numerically longer median PFS compared with patients with two or fewer events (14.0 vs. 12.0 months; p = 0.09).

**Figure 3:**
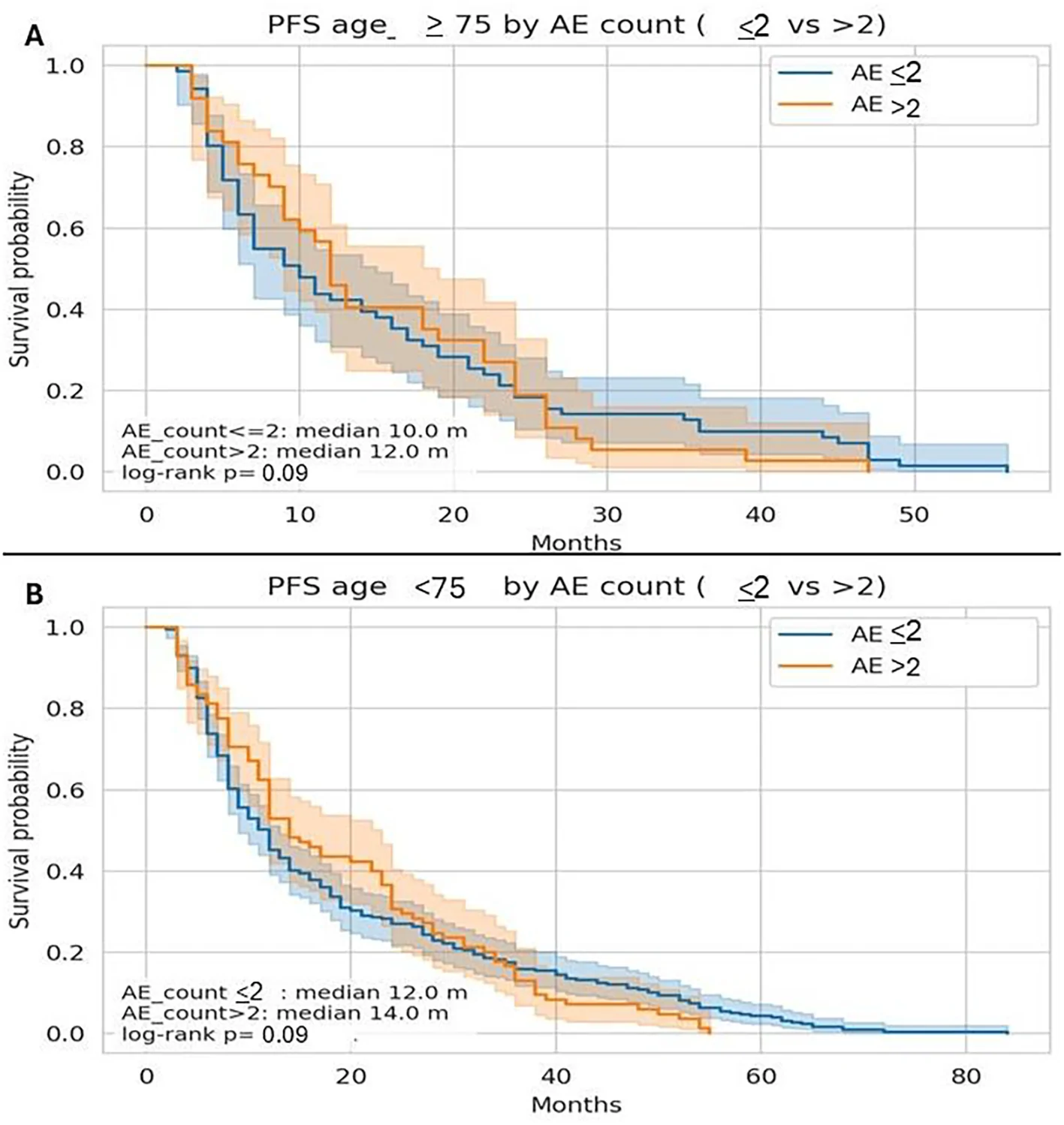
Progression-free survival (PFS) according to adverse event (AE) count (≤2 vs. >2), stratified by age (≥75 vs. <75 years). **(A)** Among patients aged ≥75 years, median PFS was 12.0 months in those with >2 AEs compared with 10.0 months in those with ≤2 AEs (p = 0.09). **(B)** Among patients aged <75 years, median PFS was 14.0 months in patients with >2 AEs versus 12.0 months in those with ≤2 AEs (p = 0.09).

Among patients <75 years (**Figure 4A**), the occurrence of at least one AE was associated with a significantly longer median OS compared to patients without adverse events (16.0 vs. 12.0 months; p = 0.045). Similarly, in patients ≥75 years of age (**Figure 4B**), the effect of AEs was even more pronounced, with median OS doubling from 8.0 to 16.0 months in the presence of an AE (p = 0.035).

**Figure 4:**
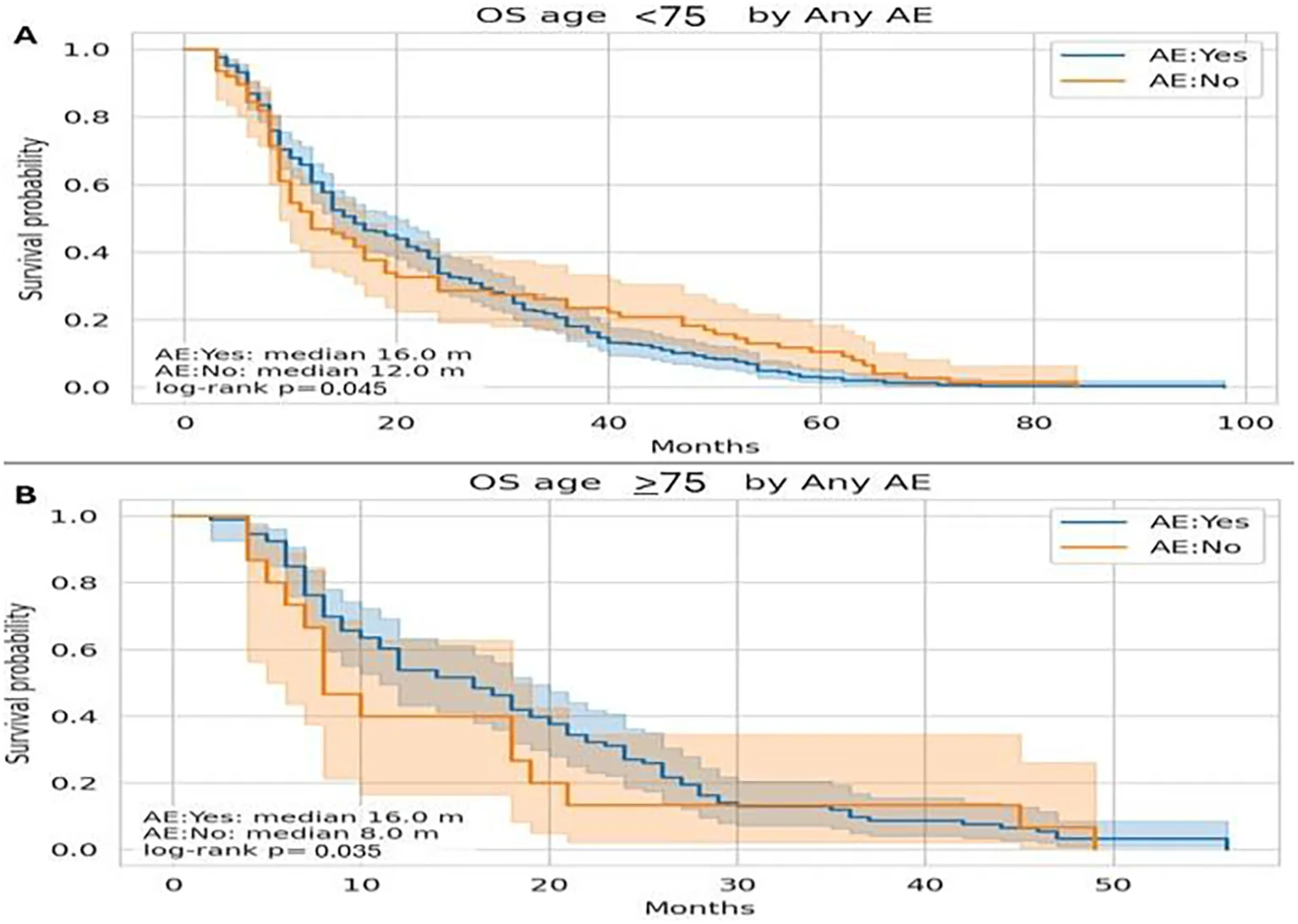
Overall survival (OS) according to the occurrence of adverse events (AEs), stratified by age (<75 vs. ≥75 years). **(A)** Among patients aged <75 years, median OS was 16.0 months in those who developed AEs compared with 12.0 months in those without AEs (p = 0.045). **(B)** Among patients aged ≥75 years, median OS was 16.0 months in patients with AEs versus 8.0 months in those without AEs (p = 0.035).

Among patients ≥75 years of age (**Figure 5A**), a higher AE count (>2 AEs) was associated with a numerical increase in median OS from 11.0 to 13.0 months, though statistical significance was not met (p = 0.08). Similarly, in patients <75 years (**Figure 5B**), experiencing a higher burden of adverse events (≤2 AEs) was associated with a longer median OS compared to those with two or fewer events (17.0 vs. 14.0 months; p = 0.05).

**Figure 5:**
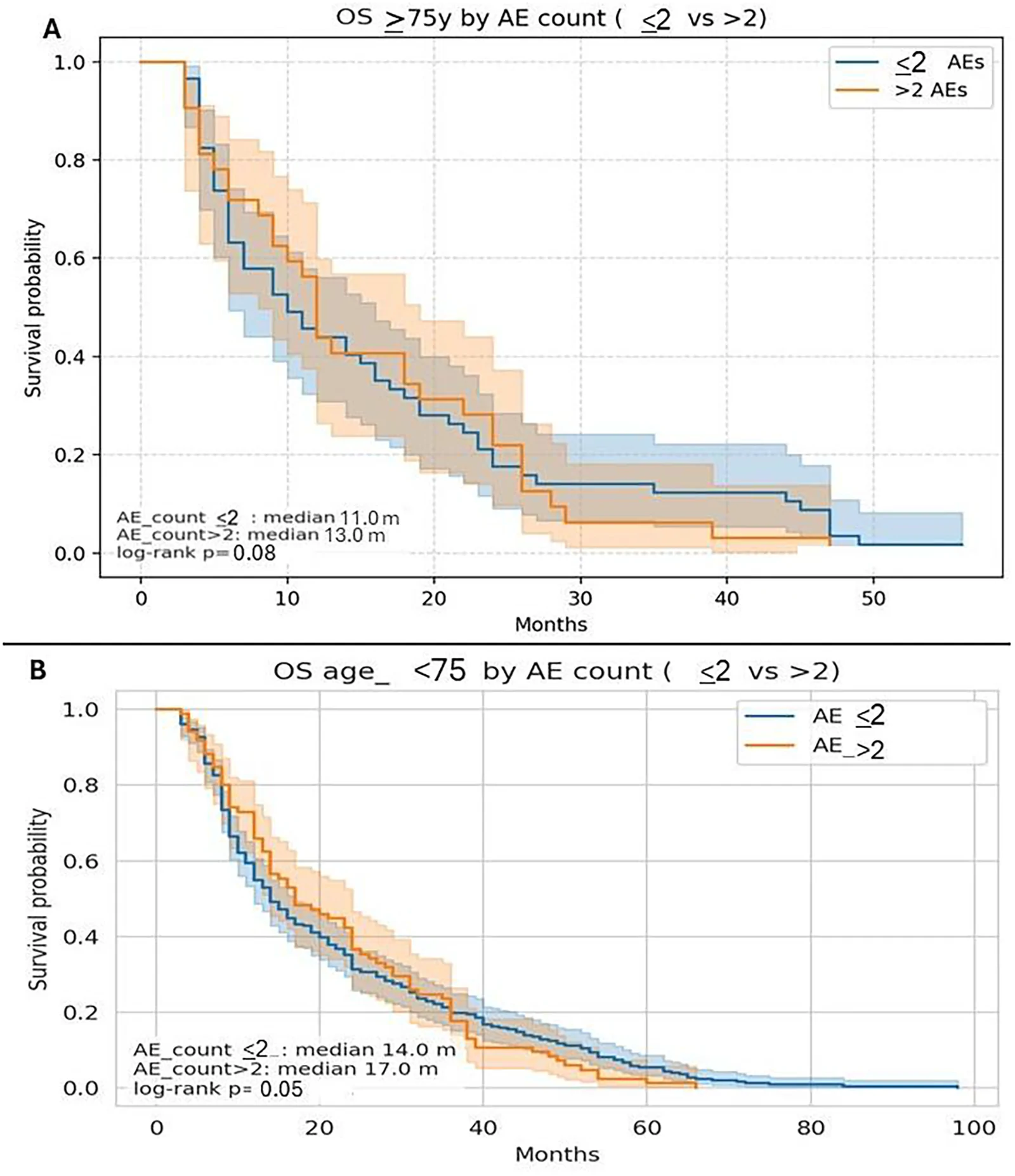
Overall survival (OS) according to adverse event (AE) count (≤ 2 vs. > 2), stratified by age (≥ 75 vs. <75 years). **(A)** Among patients ≥ 75 years of age, median OS was 13.0 months in those with > 2 AEs compared with 11.0 months in those with ≤ 2 AEs (p = 0.08). **(B)** Among patients with age <75 years, median OS was 17.0 months in patients with > 2 AEs versus 14.0 months in those with ≤ 2 AEs (p = 0.05).

Given that these comparisons were largely not statistically significant, analyses based on the number of irAEs were considered exploratory and were not interpreted as evidence of a definitive dose-response relationship between increasing irAE burden and survival.

Multivariable Cox proportional hazards analyses were performed to identify factors independently associated with PFS and OS, adjusting for age, sex, histological subtype, PD-L1 expression, smoking status, ECOG performance status, treatment type, immunotherapy regimen, and irAE occurrence (**Table 3**). Because irAEs may occur at different times after initiation of immunotherapy, irAE occurrence was incorporated into the models as a time-dependent covariate. For PFS, age ≥75 years was not independently associated with PFS (HR, 1.15; 95% CI, 0.91–1.46; P = 0.24). In contrast, ECOG performance status 2 was independently associated with shorter PFS compared with ECOG 0–1 (HR, 1.42; 95% CI, 1.08–1.86; P = 0.012). Treatment with chemo-immunotherapy was also associated with a higher risk of progression compared with immunotherapy alone (HR, 1.34; 95% CI, 1.03–1.75; P = 0.029). PD-L1 expression ≥1% showed a trend toward improved PFS, although this did not reach statistical significance (HR, 0.82; 95% CI, 0.67–1.01; P = 0.063). Notably, time-dependent irAE occurrence was independently associated with a lower risk of progression (HR, 0.72; 95% CI, 0.56–0.92; P = 0.009). For OS, age ≥75 years was independently associated with a higher risk of death (HR, 1.31; 95% CI, 1.01–1.70; P = 0.041). ECOG performance status 2 remained independently associated with inferior OS compared with ECOG 0–1 (HR, 1.55; 95% CI, 1.15–2.09; P = 0.004). Chemo-immunotherapy was associated with a numerically higher risk of death compared with immunotherapy alone, although the association did not reach statistical significance (HR, 1.29; 95% CI, 0.98–1.70; P = 0.069). Similarly, PD-L1 expression ≥1% demonstrated a trend toward improved OS (HR, 0.81; 95% CI, 0.65–1.01; P = 0.061). Time-dependent irAE occurrence was independently associated with improved OS, with a 32% lower hazard of death (HR, 0.68; 95% CI, 0.52–0.88; P = 0.004).

**Table 3:** Multivariable Cox regression analysis of PFS and OS.

Variable	PFS HR (95% CI)	OS HR (95% CI)
Age ≥75	1.15 (0.91–1.46)	1.31 (1.01–1.70)
ECOG 2	1.42 (1.08–1.86)	1.55 (1.15–2.09)
Chemo-IO	1.34 (1.03–1.75)	1.29 (0.98–1.70)
PD-L1 ≥1%	0.82 (0.67–1.01)	0.81 (0.65–1.01)
Time-dependent irAE	0.72 (0.56–0.92)	0.68 (0.52–0.88)

n, number of patients; PD-L1, Programmed Death-Ligand 1; ECOG, Eastern Cooperative Oncology Group (performance status scale); IO, Immunotherapy; Chemo-IO, Combination chemotherapy and immunotherapy; PFS, Progression-Free Survival; OS, Overall Survival; irAEs, immune-related adverse events.

## 4 Discussion

In this large real-world cohort of 452 patients with metastatic NSCLC treated with immune checkpoint inhibitor (ICI)-based regimens, we provide a comprehensive, age-stratified evaluation of treatment patterns, safety profiles, and clinical outcomes. Notably, nearly one-quarter of the study population comprised patients aged >75 years, providing valuable real-world information regarding the use of ICI-based therapy in an older population that has historically been underrepresented in pivotal randomized controlled trials. Although the age groups differed in baseline characteristics and the elderly subgroup was smaller, these factors should be considered when interpreting the age-stratified findings. Nevertheless, the inclusion of a substantial proportion of patients aged >75 years allows our study to provide clinically relevant real-world insights into the treatment patterns, tolerability, and outcomes of ICI-based therapy in this older population.

Baseline demographic and tumor characteristics were well balanced between age cohorts with respect to gender, histology, and PD-L1 expression, consistent with prior real-world evidence demonstrating that advanced chronological age alone does not fundamentally alter key baseline tumor biology in NSCLC (17, 18). Conversely, smoking patterns differed significantly, with older patients more frequently being never-smokers and less frequently active smokers. This pattern has been described previously and likely reflects generational differences in tobacco exposure rather than disease-specific biological mechanisms (19).

Older patients also demonstrated a numerical trend toward poorer performance status, with a higher prevalence of ECOG 2, consistent with population-based studies documenting the greater burden of frailty and comorbidities in elderly patients with lung cancer (20). This baseline vulnerability likely influenced treatment selection, as patients aged ≥75 years were significantly more likely to receive single-agent ICI and less likely to receive combination chemo-immunotherapy. Such age-related treatment de-escalation reflects the widespread clinical concern regarding the tolerability of cytotoxic chemotherapy in older individuals (21, 22). Furthermore, dual-agent immunotherapy with ipilimumab plus nivolumab was administered more frequently in the elderly cohort, likely reflecting a preference for chemotherapy-sparing strategies in patients considered unsuitable for platinum-based chemotherapy. This treatment approach is supported by the results of CheckMate 227 and subsequent real-world studies demonstrating the feasibility of dual immune checkpoint blockade in selected elderly patients (23, 24).

The overall incidence of irAEs was high across the cohort, mirroring real-world reports in which toxicity rates frequently exceed those observed in highly selected clinical trial populations (25). Interestingly, older patients experienced lower rates of having no irAEs (12.9% vs. 22.3%) and a higher proportion of multi-organ toxicity (>2 irAEs: 29.7% vs. 21.4%; p < 0.001). These findings challenge the traditional assumption that age-related immunosenescence protects elderly patients from autoimmune toxicity during ICI therapy. Although immunosenescence is characterized by alterations in both innate and adaptive immune function, accumulating evidence suggests that immune dysregulation accompanying aging may paradoxically predispose some older individuals to autoimmune phenomena following immune checkpoint blockade, providing a potential biological explanation for our observations (26).

When examining organ-specific toxicities, most irAE rates were comparable between age cohorts. However, arthralgia occurred significantly more frequently in patients aged ≥75 years (12.0% vs. 7.8%) consistent with previous reports describing an increased incidence of musculoskeletal toxicity among older patients receiving ICIs (26). In contrast, rates of gastrointestinal, pulmonary, hepatic, and endocrine toxicities did not differ significantly between age groups, reinforcing previous evidence that ICIs are generally manageable and well tolerated in appropriately selected elderly patients (27–29). Collectively, these findings support the growing consensus that advanced age alone should not preclude immunotherapy, provided that careful patient selection and appropriate toxicity monitoring are undertaken.

For the overall cohort, survival outcomes (median PFS: 12.0 months; median OS: 15.0 months) were consistent with contemporary real-world reports evaluating first-line ICI-based regimens in advanced NSCLC (30, 31). While progression-free survival (PFS) was comparable across age groups (12.0 vs. 11.0 months; p = 0.13), overall survival (OS) was modestly but significantly shorter in patients aged ≥75 years (14.0 vs. 15.0 months; p = 0.008). Because disease control appeared similar between the two age groups, this modest reduction in OS is more likely attributable to competing non-cancer causes of mortality, increased comorbidity burden, and a lower likelihood of receiving subsequent lines of systemic therapy after disease progression than to reduced efficacy of immunotherapy itself (32).

A central finding of this study is the robust association between irAE development and improved survival outcomes across both age groups. The occurrence of irAEs was associated with significantly prolonged PFS and OS in both younger and older patients. Importantly, among patients aged ≥75 years, the development of an irAE was associated with a doubling of median PFS (6.0 vs. 12.0 months; p = 0.05) and median OS (8.0 vs. 16.0 months; p = 0.035). These observations further support the extensive body of literature identifying irAEs as clinically meaningful surrogate markers of ICI efficacy (33–35). Mechanistically, the development of irAEs is believed to reflect enhanced immune activation and effective T-cell engagement, resulting in both autoimmune toxicity and potent anti-tumor immune responses (36). Importantly, our findings extend this concept to elderly patients, demonstrating that advanced age does not appear to diminish the biological association between immune activation and therapeutic benefit.

Our findings provide stronger support for an association between the occurrence of an irAE and clinical outcome than for a relationship between increasing irAE burden and survival. Although patients who developed irAEs generally demonstrated more favorable outcomes, comparisons according to the number of irAEs (≤2 vs. >2) were largely not statistically significant, particularly for PFS. Therefore, the observed findings should not be interpreted as demonstrating a dose-response relationship between the number of irAEs and survival. Rather, the number of irAEs should be regarded as an exploratory measure of toxicity burden. Future prospective studies incorporating the timing, duration, severity, and recurrence of individual irAEs will be required to determine whether the cumulative burden of immune-related toxicity has an independent association with clinical outcomes.

Finally, although experiencing >2 irAEs was associated with numerically longer PFS and OS compared with ≤2 irAEs, these differences did not consistently reach statistical significance. This observation is consistent with the heterogeneous findings reported in the literature. While some studies have suggested a cumulative dose-response relationship between increasing irAE burden and improved clinical outcomes, others have concluded that the occurrence of any irAE, rather than the overall number of toxicities, is the principal determinant of prognosis (37, 38). Several limitations should be considered when interpreting these findings. First, the retrospective and multicenter design introduces the possibility of selection bias, information bias, and residual confounding. Treatment selection was not randomized and differed between age groups, and therefore differences in baseline characteristics, treatment strategy, and clinical decision-making may have influenced both toxicity and survival outcomes. Although treatment type and immunotherapy regimen were incorporated into the multivariable models, residual confounding related to treatment selection cannot be completely excluded. Second, detailed information regarding baseline disease burden and metastatic distribution, particularly the presence of brain and liver metastases, was not consistently available across all participating centers. Differences in documentation practices and data availability across institutions limited our ability to reliably incorporate these variables into the multivariable analysis. Consequently, residual confounding related to the extent and distribution of metastatic disease remains possible and should be considered when interpreting the observed survival associations. Third, the timing and ascertainment of adverse events may have varied across centers because of the retrospective nature of the study. Although irAE occurrence was modeled as a time-dependent covariate to account for the timing of irAE development and to reduce potential immortal-time bias, unmeasured differences in toxicity assessment and documentation may remain. Finally, the observational nature of the study precludes establishing a causal relationship between irAE development and improved survival. The observed association should therefore be interpreted as prognostic rather than causal and requires confirmation in prospective studies with standardized collection of metastatic burden, treatment characteristics, toxicity assessment, and time-to-event data.

## 5 Conclusion

In this multicenter retrospective cohort, the occurrence of irAEs was associated with more favorable progression-free survival and overall survival among patients with advanced NSCLC receiving ICI-based therapy, including those aged 75 years and older. These findings support the use of ICIs in carefully selected elderly patients and suggest that age alone should not preclude immunotherapy. The association between irAE occurrence and improved outcomes further suggests that irAEs may serve as a clinically relevant marker of treatment benefit in this population. However, the exploratory analysis of irAE burden did not demonstrate a consistent dose-response relationship, and a greater number of irAEs should therefore not be interpreted as directly improving survival. Prospective studies incorporating comprehensive geriatric assessments and immune biomarkers are warranted to better define predictors of ICI benefit and toxicity and to optimize patient selection and treatment outcomes in elderly patients with advanced NSCLC.

## Data Availability

The raw data supporting the conclusions of this article will be made available by the authors, without undue reservation.
